# Photovaporisation prostatique au laser chez les patients à haut risque hémorragique

**DOI:** 10.11604/pamj.2013.16.2.2853

**Published:** 2013-09-03

**Authors:** Zakaria Bouabdallah, Amine Kharbouchi, Alexandre Colau, Gerard Cariou

**Affiliations:** 1 Service d'urologie, groupe hospitalier Diaconesses Croix Saint-Simon, Paris, France

**Keywords:** Photovaporisation, prostate, laser, hémorragie, Photovaporisation, prostate, laser, hemorrhage

## Abstract

**Introduction:**

Les patients sous traitement anticoagulant sont à risque élevé de saignement lors de la résection transurétrale de la prostate ou de l'adénomectomie par taille vésicale et ils se voient souvent récuser pour la chirurgie de l'hyperplasie bénigne de la prostate symptomatique. En Utilisant la photovaporisation de la prostate, les patients à haut risque peuvent subir en toute sécurité la chirurgie. Nous avons évalué l'innocuité et l'efficacité de la photovaporisation de la prostate (PVP) chez les patients sous anticoagulants en cours avec les dérivés de la coumarine, l'aspirine ou le clopidogrel, se plaignant de symptômes d'hypertrophie bénigne de la prostate.

**Méthodes:**

Entre janvier 2009 et mai 2010, 47 hommes sous anticoagulation systémique ont subi une photovaporisation de la prostate. Les données ont été recueillies sur les caractéristiques démographiques, les comorbidités, les complications, la natrémie, l'hémoglobine, le débit urinaire maximal, le résidu post-mictionnel, l'IPSS et les complications.

**Résultats:**

L'âge moyen était de 78 ans, le volume prostatique moyen était de 44g et le PSA était de 3.4ng/ml. Parmi les 10 patients (21.2%) étaient sous AVK, 27 (57.4%) étaient sous aspirine, 2 (4.2%) étaient sous clopidogrel, un sous fondaparinux et 6 (12.7%) étaient sous 2 anticoagulants ou plus. Le score ASA moyen était de 3. La durée moyenne de fonctionnement de l'appareil était de 38 minutes, l'énergie moyenne utilisée était de 200kJ. La durée moyenne d'hospitalisation était de 2 jours. Les complications survenant dans les 30 jours comprenaient une infection urinaire chez 5 patients (10.6%), une dysurie chez 4 patients et une hémorragie retardée chez 4 autres (8.5%). Un seul de ces patients a nécessité une transfusion sanguine et aucun patient n'a nécessité une réintervention. En 3 mois de suivi un seul patient a nécessité une incision du col vésical pour sclérose du col. Aucune incontinence ou sténose urétrale n'a été rapportée. Des améliorations significatives ont été notées dans l'IPSS, le débit urinaire maximal et le résidu post-mictionnel.

**Conclusion:**

La PVP est caractérisé par d'excellentes propriétés hémostatiques et taux très faible de complications peropératoires même chez les patients sous 2 ou plusieurs agents anticoagulants. Sur la base de nos résultats péri-opératoires, nous recommandons la PVP comme traitement chirurgical de première intention chez les patients à haut risque de hémorragique souffrant de symptômes d'hypertrophie bénigne de la prostate.

## Introduction

Environ la moitié des hommes de plus de 60 ans ont souvent des symptômes du bas appareil urinaire en rapport avec l′hypertrophie bénigne de la prostate [1]. Depuis quelques années nous assistons à une généralisation de la prescription des traitements anti thrombotiques au long cours. Cette situation pose de nombreux problèmes lorsqu′une prise en charge chirurgicale est indiquée chez ces patients qu′elle soit urgente ou même programmée [2].

La chirurgie de l′HBP est particulièrement concernée par cette problématique, car c′est une chirurgie très hémorragique et le caractère essentiellement fonctionnel de ses indications complique la résolution de l′équation bénéfices-risques, surtout chez le patient sous traitement anti coagulant.

La question de la technique chirurgicale la plus adaptée doit également se poser: cette dernière doit permettre de limiter au maximum les risques hémorragiques et autoriser une reprise rapide de l′anticoagulant en postopératoire.

La résection trans-urétral de la prostate (RTUP) est le traitement chirurgical de référence de l′HBP [3]. Néanmoins, sa morbidité hémorragique n′est pas négligeable [4–6]. Des études récentes ont montré que les taux de transfusion sanguine étaient de l′ordre de 2 à 7.1% avec des taux de reprise précoce de 3-5% [6]. Ainsi la RTUP chez les patients sous traitement anticoagulant oral ou avec des troubles de la coagulation est contre-indiquée.

Afin de réduire la morbidité et les complications associées à la RTUP, les techniques chirurgicales au laser ont été introduites durant la dernière décennie [7, 8]. La photo vaporisation sélective de la prostate (PVP) avec le laser KTP a été introduite comme traitement de substitution chez les patients présentant des troubles du bas appareil urinaire. Elle est supposée offrir aux patients une thérapie plus efficace avec une plus grande sécurité que la RTUP [9–14]. Elle combine les propriétés de réduction du volume tissulaire de la RTUP avec le profil d′innocuité de la chirurgie au laser. La puissance maximale du laser KTP a été augmentée au fil du temps pour minimiser le risque opératoire lié à la vaporisation des grandes glandes [11]. Bien que de nombreuses études ont montré que le GreenLight 120-W HPS est efficace et sans danger pour les patients atteints d′HBP [15–17], son utilisation chez les patients atteints d′HBP mais avec un risque élevé de saignement a été relativement peu rapportée [18–20].

Le laser KTP pourrait-il être une option thérapeutique appropriée chez les patients sous anti thrombotiques ′ Pour tenter de répondre à cette question, nous avons proposé une étude rétrospective dont l′objectif était de vérifier la faisabilité de cette technique chez des patients sous anticoagulants et de relever le taux de complications et la morbidité.

## Méthodes

De janvier 2009 à mai 2010, un total de 169 patients ayant une hypertrophie bénigne de prostate avec un haut risque cardiovasculaire et sous anticoagulation à long terme ont subi une photo-vaporisation prostatique au laser GreenLight HPS 120-W pour des troubles du bas appareil urinaire secondaires à l′HBP. Notre définition du risque élevé était un score de la société américaine d′anesthésiologie (ASA) supérieur ou égal à 3 évalué par un anesthésiste de l′équipe [21]. Parmi ces patients, 47 prenaient un traitement anticoagulant oral, et son retrait les aurait exposés à un risque considérable d′événements thromboemboliques.

L′évaluation préopératoire des patients a compris les antécédents médicaux, un examen physique avec les données du toucher rectal, une échographie rénale et pelvienne avec une évaluation du résidu post-mictionnel, une débitmètrie, une analyse d′urines de routine, une analyse de sang avec les paramètres de la coagulation, une évaluation des fonctions cardiaque et pulmonaire et la détermination du taux sérique de l′antigène spécifique de la prostate (PSA). Tous les patients ont rempli le score international des symptômes de prostatisme (I-PSS).

Les critères d′inclusion étaient tous les patients avec une HBP symptomatique échappant au traitement médical bien conduit et pour lesquels une indication opératoire était retenue du fait de l′importance de la symptomatologie avec un débit urinaire maximal (Qmax) de moins de 15 ml/s ou un RPM de plus de 100ml ou un I-PSS de 8 ou plus. Les patients atteints de cancer de prostate ou de vessie et les hommes présentant des signes du bas appareil urinaire non liés à l′HBP (vessie neurologique, instabilité vésicale) ont été exclus de l′étude.

Certains patients avaient un cathéter (sus-pubien ou trans-urétral) avant l′intervention du fait de la rétention urinaire.

Tous les patients ont donné leur consentement éclairé. Les patients ont été évalués en préopératoire, à 1mois et puis à 3mois après l′intervention. La photovaporisation prostatique était réalisée par les six urologues du service.

En l′absence de vrai consensus et en concertation avec les médecins anesthésistes de l′équipe, une homogénéisation des pratiques vis-à-vis de la poursuite ou non de ces traitements pour l′acte chirurgical a été proposé pour tous nos patients sous traitement anti thrombotique. Le choix du type d′anesthésie se faisait en fonction du terrain et de l′anticoagulant utilisé. En cas d′anesthésie générale l′anticoagulant n′était pas arrêté alors qu′en cas d′anesthésie locorégionale l′anticoagulant était arrêté à J-5 et repris le soir même ou le lendemain. A l′arrêt des anti-vitamines K (AVK) le relais se faisait par des doses curatives d′héparine de bas poids moléculaire (HBPM) administrée par vois sous cutanée, démarrée 24h après l′interruption des AVK et arrêtée 12h avant la chirurgie quand l′INR baisse au-dessous de 1,4 puis repris 12h après le geste. L′AVK est ensuite repris dès que le patient est capable de reprendre une alimentation et quand l′INR augmentait au-dessus de 2 l′HBPM pouvait être arrêté.

La photovaporisation prostatique a été réalisée à l′aide d′un laser HPS KTP 120-W fourni par un système GreenLight HPS, émettant une lumière verte avec une longueur d′onde de 532 nm. Un cystoscope 30° 23-F à flux continu a été utilisé avec le laser réglé à 80 W d′abord, 120 W pour la vaporisation, et 30-40 W pour la coagulation. La fibre est insérée dans le cystoscope laser à flux continu avec un canal d′irrigation séparé. L′eau stérile à température ambiante a été utilisée pour l′irrigation. Une configuration permettant un flux continu assurait une visibilité et un refroidissement suffisamment de la fibre.

Le système laser fournit un faisceau de visée rouge pointant vers la direction réelle du faisceau laser KTP. La vaporisation de la prostate a été réalisée sous vision directe en utilisant la fibre laser avec une technique de balayage de contact. Pour atteindre une vaporisation efficace du tissu, une faible distance de travail d′environ 2 mm est maintenue. La procédure est réalisée comme une RTUP, en commençant par le col de la vessie et les lobes latéraux, le lobe antérieur, et enfin la partie apicale de la prostate [20]. Après la fin de la vaporisation au niveau des fibres capsulaires, une cavité dont la forme ressemble à celle de la RTUP est obtenue.

Le sondage vésical en fin d′intervention n′était pas systématique. La décision dépendait du volume de la prostate, de la quantité d′énergie délivrée et de la fonction vésicale. En cas de sondage une sonde double courant charrière 20 était utilisée. L′irrigation avec une solution saline est débutée dans le bloc opératoire. Lorsque les patients ont récupéré de l′anesthésie, ils pouvaient boire suffisamment, et lorsque leur urine était redevenue claire, l′irrigation était arrêtée. Une numération formule sanguine et les taux sériques de sodium ont été mesurés le soir même après retour au service. Si les urines étaient claires et en l′absence de complications périopératoires une tentative d′ablation de la sonde vésicale était réalisée dès le lendemain de la chirurgie, le cas échéant le sondage était prolongé. En l′absence de reprise de mictions, une sonde vésicale a été réinsérée.

Un ECBU était systématiquement réalisé à l′ablation de la sonde. Un taux d′hémoglobine post-opératoire inférieur à 10ng/ml donnait lieu à une transfusion sanguine.

En peropératoire, ont été notés la durée de l′intervention et la quantité d′énergie laser délivrée. En post-opératoire, ont été recueillies la survenue d′un cailloutage vésical au cours de l′hospitalisation et nécessitant une intervention d′un médecin (avec ou sans reprise chirurgicale), une transfusion sanguine, une rétention urinaire survenant après retrait du cathéter, les événements thromboemboliques (thrombose veineuse profonde ou embolie pulmonaire), une hématurie survenant après la sortie du patient, une infection, une sténose urétrale et une consultation aux urgences ou un décès de toute cause dans les 3 mois suivant l′intervention.

## Résultats

Des 169 patients traités par photo vaporisation prostatique au laser Green Light^®^ HPS entre janvier 2009 et mai 2010, 47 avaient un risque hémorragique élevé en raison d′un traitement anticoagulant au long cours. Parmi ces patients 21.2% (n = 10) étaient sous anti vitamine K, 57.4% (n = 27) sous aspirine, 4.2% (n = 2), sous clopidogrel, 12.7% (n = 6) sous association clopidogrel et aspirine, un seul patient sous association AVK et aspirine, et un autre sous fondaparinux. Le Tableau 1 énumère les principales raisons de l′anticoagulation. L′âge moyen des patients sous anticoagulants était de 78±;8 ans (55-85), le score ASA moyen était de 2,5 ± 0,5 (1-4). 10 patients avaient une histoire d′infarctus du myocarde, une arythmie cardiaque était présente chez 6 patients, 5 patients avaient une valvulopathie, un ATCD d′accident vasculaire cérébral a été noté chez 7 patients et 6 patients avaient une artériopathie des membres inférieurs. Les dossiers ne contenaient pas la cause du traitement anticoagulant pour 13 autres patients.

**Tableau 1:** Principales causes du traitement anticoagulant

Comorbidités	Nombre de patients
Infarctus du myocarde	10
valvulopathie	5
arythmie	6
AVC	7
Artériopathie des membres inférieurs	6
Raison inconnue	13

Le Tableau 2 présente les paramètres périopératoires. Avant la chirurgie 21.2% (n = 10) des patients avaient un cathéter à demeure à cause d′une rétention urinaire réfractaire. L′anesthésie loco-régionale a été réalisée chez 46.8% (n = 22) avec un arrêt de l′anticoagulant à J-5 et une reprise le soir même ou le lendemain, et l′anesthésie générale chez 53.2% (n = 25) des patients.

**Tableau 2:** Paramètres périopératoires

**Nombre de patients**	47
Age	78(55-85)
Volume prostatique (g)	44 (20-80)
PSA (ng/ml)	3.4
INR	1.45
Durée opératoire	38 (15-70)
Energie délivrée (kj)	200 (20-280)
Liquide d'irrigation (L)	12
Durée d'hospitalisation en jours	2 (1-6)

En cas d′anesthésie générale l′anticoagulant n′était pas arrêté, cela était le cas de 19 patients sous aspirine, de 2 autres patients sous clopidogrel, de 3 patients sous AVK avec un INR compris entre 2 et 2.5, et du seul patient sous fondaparinux. La durée moyenne de l′opération était 38minutes (15 à 70min) avec une énergie moyenne délivrée de 200kj (20 à 280).

Chez les patients traités par AVK le taux de prothrombine moyen préopératoire était de 35% (20 - 59%), ce qui équivaut à un INR (ratio international normalisé) de 2.1 (1.3 à 2.9). Un seul saignement peropératoire cliniquement significatif a été observé chez un patient sous AVK ayant nécessité une conversion en RTUP, et une seule transfusion sanguine a été nécessaire à j2 chez un patient sous aspirine.

Les taux moyens d′hémoglobine préopératoire et post-opératoire étaient de 12.3g/dl (9.2 à 14.5) et 11 g/dl (8.6 à 12.1) respectivement. Malgré le changement des valeurs d′hémoglobine après la procédure, ce changement n′était pas cliniquement significatif. Par ailleurs, aucun patient n′a montré des signes de TURP syndrome.

Chez nos patients le taux de sodium sérique préopératoire moyen était de 138mmol / L (extrêmes: 131-145), et le taux moyen postopératoire immédiat était de 137mmol / L (extrêmes: 130-143). L′évaluation des taux de sodium sériques en pré et post opératoire n′a pas montré de différence significative. 14.8% (n = 7) des patients n′ont pas été sondés en fin d′intervention.

En raison d′une légère hématurie, 76.6% (n = 36) des patients ont reçu une irrigation vésicale postopératoire pendant 24 heures. Particulièrement les patients recevant des AVK avec un INR> 2.0 ont eu une irrigation post-opératoire, en règle pendant 24 h. Chez ces patients, le temps de cathétérisme était plus long.

La durée moyenne d′hospitalisation post-opératoire était de 2 jours (1-6 jours) (Tableau 2). Outre une hématurie transitoire, l′autre raison du retrait retardé du cathéter et d′une hospitalisation prolongée était une morbidité importante d′ù le besoin de plus de temps pour récupérer après la chirurgie. Un seul patient âgé de 83ans ASA III est décédé subitement à j2 post-opératoire d′une cause inexpliquée. Aucune autre admission en unité de soins intensifs pour problèmes cardiaques (infarctus du myocarde) n′a été relevée durant l′étude.

Parmi nos patients, 3 ont nécessité un resondage en post-opératoire en raison de la survenue d′une rétention urinaire après ablation de la sonde. Chez ces patients, le cathéter trans-urétral a été retiré 3-5 jours plus tard.

Dans la période postopératoire précoce (< 30 j) une légère dysurie et une pollakiurie transitoires ont été observées chez 8,5% (n = 4) des patients. Ces symptômes ont été traités avec succès par l′administration de médicaments anti-inflammatoires non stéroïdiens pendant quelques jours. Une culture d′urines positives indiquant une infection urinaire a été détectée dans 10.6% (n = 5) des cas.

Au cours des 3mois suivant l′intervention nous avons observé une sclérose du col vésical chez un seul patient. Il n′a été observé aucun cas de sténose de l′urètre ou d′incontinence urinaire d′effort. Les événements indésirables postopératoires sont regroupés dans le Tableau 3. Le Tableau 4 montre les paramètres mictionnels subjectifs et objectifs au cours du suivi des patients. Une amélioration significative des IPSS, de la qualité de vie, du Qmax, et du RPM a été observée et maintenue au cours de la période d′observation allant jusqu′à 3 mois.

**Tableau 3:** Événementsindésirablespostopératoires

**Nombre de patients**	47
**Précoces**	
Rétention	3
Transfusion	1
Hématurie	4
Dysurie -pollakiurie	4
Infection urinaire	5
**Tardifs**	
Sclérose du col vésical	1
Sténose de l'urètre	0
Ré-intervention	0
incontinence	0

**Tableau 4:** Paramètres mictionnels avant et après la PVP

	IPSS	Qualité de vie	Qmax (ml/s)	RPM (ml)
**Avant**	18.2±6.3	3.7±1.4	7.9±5	190±112
**A 3 mois**	7.3±4.9	1.2±1.3	20±10.5	24±30

## Discussion

L′HBP est l′étiologie la plus fréquente des TUBA chez les personnes âgées et elle est la pathologie la plus fréquente nécessitant un traitement chirurgical chez les hommes. L′utilisation accrue du traitement médical de l′HBP retarde le recours à la chirurgie ce qui conduit à un groupe de traitement avec plus de comorbidités. Une Interruption de long terme de l′anticoagulation chez ces patients crée une situation complexe dans laquelle des risques concurrents de thrombose et d′hémorragie doivent être géré [6].

Parmi les techniques chirurgicales, la RTUP est toujours considéré comme le gold standard pour le traitement de l′HBP, mais son principal inconvénient est représenté par les pertes sanguines même si elle améliore significativement les symptômes urinaires et le flux urinaire. En raison du risque hémorragique accru, un traitement anticoagulant en cours est ainsi une contre-indication stricte pour la RTUP.

À ce jour, il n′existe pas de consensus sur la prise en charge appropriée péri-opératoire de l′anticoagulation chez les patients traités au long cours par les dérivés de la coumarine. La procédure la plus couramment utilisée est l′arrêt du traitement anticoagulant pour un minimum de 4 jours avant l′opération et l′utilisation d′un relais avec héparine par voie injectable pour réduire la durée de la période sans anticoagulant.

Chakravarti et al. ont géré l′anticoagulation chez 11 patients qui ont subi une RTUP en arrêtant la warfarine et en démarrant l′héparine intraveineuse non fractionnée 2 jours avant le geste. Ils ont eu un seul cas ù ils ont eu recours à une transfusion sanguine, mais 27% des patients ont été réadmis à l′hôpital en raison de complications hémorragiques mineures [22].

Dotan et al. [23] ont étudié l′utilisation d′HBPM comme thérapie de relais chez 20 patients sous warfarine, parmi eux 20% ont nécessité des transfusions sanguines et 10% avaient des saignements post-opératoire précoces qui ont conduit à un resondage, contribuant ainsi à un séjour prolongé à l′hôpital.

Parr et al sur une série de 13 RTUP et une résection trans-urétrale de la vessie (RTUV) chez des patients sous anticoagulation orale sans interruption ont rapporté un taux de transfusion sanguine de 30% et un taux de transfusion de plasma frais congelé de 50% [24].

Descazeaud et al ont examiné l′impact de l′anticoagulation orale sur 612 patients qui ont subi une RTUP, dont 206 étaient sous anticoagulation en préopératoire [25]. Tous les patients ont arrêté l′anticoagulation avant l′intervention chirurgicale avec un INR préopératoire inférieur à 1,5. Les patients du groupe traitement anticoagulant ont eu un séjour hospitalier nettement plus long, des taux plus élevés de transfusion sanguine, d′hématurie et d′événements thromboemboliques. D′autre part, un simple retrait péri opératoire des anticoagulants, sans aucune substitution implique un risque thromboembolique certain lié au terrain.

En résumé, les résultats des différentes stratégies semblent conduire à une baisse des fréquences des complications liées à la thrombose, mais la hausse des taux de saignement post-opératoire, en particulier avec les stratégies d′anticoagulation plus agressives. Ainsi la RTUP chez le patient sous anticoagulants est un exercice de marche sur une corde entre le risque accru de saignement et d′événements thromboemboliques.

Actuellement, il existe un certain nombre de procédures minimalement invasives qui peuvent être des alternatives sûres et efficaces à la RTUP. Ainsi au cours des 10-15 dernières années, une variété de techniques endoscopiques au laser a été évaluée pour le traitement de l′HBP et qui ont été associées à une morbidité moindre que la RTUP avec une hospitalisation plus courte.

Dans une étude japonaise, Okamura et al ont comparé les résultats de la RTUP chez 3918 patients, de la résection transurétrale dans une solution de chlorure de sodium chez 242 autres, l′énucléation de la prostate au laser holmium chez 638, la vaporésection de la prostate au laser holmium (HoLAP) chez 90 et la PVP chez 241 patients [26]. Deux patients (0.05%) qui sont subi une RTUP sont morts. Le taux de transfusion était de 25.6% pour la résection transurétrale dans une solution saline, 10.2% pour la RTUP et de 7.8% pour l′énucléation de la prostate au laser holmium. Aucun patient dans le groupe HoLAP ou PVP n′a nécessité de transfusion sanguine, mais il y avait une proportion significativement plus élevée de patients sous warfarine dans ces groupes (p < ; 0.01). Globalement, il semble y avoir beaucoup moins de morbidité dans la PVP et le HOLAP que dans la RTUP. La diminution des pertes sanguines et moins de changements hémodynamiques sont parmi les avantages les plus importants de la PVP laser par rapport à la RTUP traditionnelle.

Il a été démontré pour le laser KTP une vaporisation du tissu prostatique avec une coagulation minimale des structures sous-jacentes et que la chaleur était concentrée dans un petit volume, ce qui provoquait l′ablation du tissu par vaporisation rapide de l′eau cellulaire en laissant une couche de 2-mm de tissu coagulé. Les études in vivo ont montré que les grandes zones de coagulation pendant la vaporisation au laser KTP 80 W rendent cette technique une procédure ablative relativement exsangue, donnant lieu à une hémostase, qui est très supérieure à la RTUP conventionnelle [27].

La réduction de la morbidité (saignement ou autres troubles cardiaques majeurs) et une hospitalisation plus courte ont été signalés comme les facteurs conduisant à l′acceptation rapide de la PVP [26].

Reich et al ont rapporté sur 66 patients avec une taille de la prostate moyenne de 49 g et un score ASA de 3 ou plus ayant subi une PVP [28]. Parmi ces 29 patients (44%) étaient sous anticoagulants oraux ou avaient une diathèse hémorragique. Ils n′ont pas noté de complications péri-opératoires et n′ont eu recours à aucune transfusion sanguine. L′énergie moyenne du laser délivrée était de 156 kJ. Des améliorations significatives ont été relevées dans l′I-PSS, le RPM et le Qmax jusqu′à 12 mois de suivi, comme c′est le cas dans notre étude [28].

Notre étude montre que la PVP chez les patients sous anticoagulation systémique peut être sûre et efficace. Le score ASA moyen élevé reflète le nombre important de patients morbides qui ont subi avec succès une PVP en toute sécurité.

Ruszat et al ont comparé les résultats de 116 cas sous anticoagulants et de 92 contrôles traités par PVP. Les 2 groupes étaient similaires en terme de volume prostatique moyen (62 vs 57 cc), de durée opératoire (67± 28 vs 63± 29 minutes) et de l′énergie laser utilisée (221 vs 210 kJ). Aucune rétention urinaire par caillots n′a été notée et aucune transfusion sanguine n′a été nécessaire après l′opération. À 24 mois, le taux de complications à long terme était faible dans le groupe anticoagulant et le groupe témoin (13% et 15%, respectivement) [19].

Yuan et al. ont rapporté les résultats de 12 mois d′un essai clinique prospectif de 128 hommes à haut risque avec une hyperplasie bénigne de la prostate ayant subi une PVP (23). Selon leurs résultats, la durée de fonctionnement moyenne était de 51.6 min, le temps de cathétérisme moyen était de 2.8 jours, le score IPSS a diminué, passant de 19.2 avant la chirurgie à 6.1, le Q (max) est passé de 7.0 ml/s à 24.8 ml/s, et de l′urine résiduelle est passée de 168 mL à 23 ml, 12 mois plus tard. Tous ces résultats sont similaires à nos résultats cliniques [29].

Karatas et al. sur une série de 67 patients cardiaques à haut risque ayant subi une PVP ont noté une diminution du volume prostatique moyen de 53% à 6 mois avec une amélioration significative des paramètres urinaires [30].

D′autre part, Ruszat et al. et Woo et al. ont indiqué qu′il n′y avait pas d′arrêt péri-opératoire dans l′administration du médicament dès que son retrait aurait posé un risque considérable d′événements thromboemboliques [19, 31]. Nous n′avons pas arrêté l′anticoagulant dans certains cas et l′arrêter dans d′autres cas 5 jours avant le geste avec un relais par des HBPM et une reprise le soir ou le lendemain de l′intervention. Nous n′avons pas observé de complications hémorragiques majeures ou thromboemboliques liées à la gestion du traitement anticoagulant.

La nouvelle génération de lasers KTP à haute puissance peut être utilisée dans le cadre de soins de jour, avec un risque minime de complications et sans la nécessité de drainage par cathéter post-opératoire ce qui a été les cas chez 7 de nos patients [27, 30, 32]. Plusieurs équipes ont rapportés cette donnée notamment Hai et Malek qui n′ont pas posé de sonde postopératoire dans 20% des cas [33].

Dans les derniers articles publiés la durée de sondage restait inférieure à 24 heures [18, 34]. En outre, la possibilité d′utiliser cette technologie dans la gestion de prostates de volume relativement important, d′une manière sûre et efficace, représente un autre avantage de cette technique, ce qui la rend préférable aux autres solutions chirurgicales, y compris l′adénomectomie par taille ou la RTUP.

Les complications peropératoires ont été rares. Nous avons eu recours à une seule conversion en RTUP en début d′expérience. Ceci est confirmé par les principales séries de la littérature. Le taux de complications postopératoires rejoint les résultats rapportés par la littérature. Cependant leur rareté conforte l′intérêt du laser KTP chez les patients sous traitements anticoagulants [19, 27, 31].

En se basant sur nos données, nous suggérons que les lasers KTP de forte puissance représentent un défi très important pour le statut du «;gold standard»; de la RTUP. En effet la PVP laser a été jugée suffisamment efficace pour nos patients recevant un traitement anticoagulant en raison de certains problèmes cardio-vasculaires. Ainsi des améliorations significatives similaires à celles produites par la RTUP ont été notées chez la majorité des patients à la fois dans les scores de symptômes IPSS mais aussi les débits maximaux et les volumes résiduels. Quelques complications limitées ont été observées et en dehors de la dysurie légère et l′urgenturie aucune complication majeure qui affecte vraiment la qualité de vie des patients traités n′a été observée dans notre série. Le cathéter urétral a été enlevé dans les 48 heures chez l′immense majorité de nos patients et la plupart des patients sont sortis le jour suivant. Plus important encore, même si une hématurie discrète a été observée durant quelques jours après l′intervention chez certains patients, aucune hémorragie sévère nécessitant une intervention n′a été observée ce qui confirme l′efficacité hémostatique de la vaporisation au laser KTP avec une résection de tissus très efficace surtout chez les patients à haut risque en donnant lieu à une hémostase efficace.

Malgré tous ces bons résultats, il faut souligner que le nombre relativement restreint de patients et la courte période de suivi avec l′absence d′un groupe témoin pourrait être considéré comme le point faible de notre étude.

## Conclusion

La photovaporisation prostatique au laser Greenlight 120-W chez des patients à haut risque hémorragique en raison d′une anticoagulation orale par aspirine, anti-vitamines K, clopidogrel, fondaparinux ou même ceux sous 2 agents anticoagulants peut être réalisée en toute sécurité même pour une grosse prostate nécessitant une intervention de plus longue durée sans risque accru de saignement péri-opératoire avec peu de complications et une amélioration clinique significative et durable. Ainsi, en offrant une ablation exsangue et immédiate du tissu prostatique, la PVP peut être considéré comme une alternative très sérieuse à la RTUP chez tous les patients souffrant de troubles sévères du bas appareil urinaire secondaires à l′hypertrophie bénigne de la prostate et comme traitement de première intention chez les patients sous anticoagulation orale en cours. Des interrogations subsistent encore sur les modalités de gestion du traitement anticoagulant, mais à terme il apparait indispensable de définir une attitude claire et systématique. Tout comme il serait judicieux de confirmer ces résultats en les comparant à ceux de la RTUP d′ù l′intérêt de réaliser des essais cliniques prospectifs randomisés avec des données de suivi à long terme afin de déterminer son efficacité, son innocuité et la durabilité du résultat obtenu.
